# Protective Effects of Omega-3 Supplementation against Doxorubicin-Induced Deleterious Effects on the Liver and Kidneys of Rats

**DOI:** 10.3390/molecules28073004

**Published:** 2023-03-28

**Authors:** Sara Gomes Espírito Santo, Marina Gaiato Monte, Bertha Furlan Polegato, Luís Fernando Barbisan, Guilherme Ribeiro Romualdo

**Affiliations:** 1 Department of Pathology, Botucatu Medical School, São Paulo State University (UNESP), Botucatu 18618687, SP, Brazil; 2 Internal Medicine Department, Botucatu Medical School, São Paulo State University (UNESP), Botucatu 18618687, SP, Brazil; 3 Department of Structural and Functional Biology, Institute of Biosciences of Botucatu, São Paulo State University (UNESP), Botucatu 18618689, SP, Brazil

**Keywords:** cancer treatment, doxorubicin, doxorubicin side effects, omega 3, hepatotoxicity, nephrotoxicity, genotoxicity, Wistar rats

## Abstract

Anthracycline doxorubicin (DOX) is still widely used as a chemotherapeutic drug for some solid tumors. Although DOX is highly effective, its side effects are limiting factors, such as cardio, nephro and hepatotoxicity. As such, approaches used to mitigate these adverse effects are highly encouraged. Omega 3 (ω-3), which is a class of long-chain polyunsaturated fatty acids, has been shown to have anti-inflammatory and antioxidant effects in preclinical bioassays. Thus, we evaluated the protective effects of ω-3 supplementation on hepatotoxicity and nephrotoxicity induced by multiple DOX administrations in rodents. Male Wistar rats (10 rats/group) were treated daily with ω-3 (400 mg/kg/day) by gavage for six weeks. Two weeks after the first ω-3 administration, the rats received DOX (3.5 mg/kg, intraperitoneal, 1×/week) for four weeks. DOX treatment reduced body weight gain increased systemic genotoxicity and caused liver-related (increase in serum ALT levels, thickness of the Glisson’s capsule, compensatory proliferation and p65 levels) and kidney-related (increase in serum urea and creatinine levels, and incidence of tubular dilatation) deleterious outcomes. In contrast, ω-3 supplementation was safe and abrogated the DOX-related enhancement of systemic genotoxicity, serum urea and creatinine levels. Furthermore, ω-3 intervention reduced by 50% the incidence of kidney histological lesions while reducing by 40–50% the p65 protein level, and the proliferative response in the liver induced by DOX. Our findings indicate that ω-3 intervention attenuated the DOX-induced deleterious effects in the liver and kidney. Therefore, our findings may inspire future mechanistical investigations and clinical interventions with ω-3 on the reported outcomes.

## 1. Introduction

According to the World Health Organization (WHO), in 2019, cancer was ranked as the leading cause of death before 70 in several countries, with an estimated 19.3 million new cases and 10 million cancer deaths worldwide in 2020 [1,2]. In contrast, the development of different oncological therapies, including chemotherapy, is generally linked to a better quality of life and increased survival [3]. Chemotherapy is considered an advance in cancer treatment, but side effects are still a limiting barrier to the efficacy of this clinical intervention [3]. Doxorubicin (DOX), an anthracycline produced by *Streptomyces peucetius* bacterium, has been widely used since the 1970s as a chemotherapeutic drug for solid tumors (ovary, breast and gastrointestinal) due to its high efficacy [4,5]. The antitumor effect of DOX is related to the drug’s interaction with cellular DNA, intercalating to the double strand and inhibiting repair mediated by the enzyme topoisomerase 2. DOX also causes mitochondrial dysfunction, which leads to the formation of free radicals, which can damage the membranes, nucleic acids and proteins of the tumor cells. The subsequent activation of cell death pathways also contributes to the antitumoral effect of this anthracycline [5].

Although they are highly effective against cancer, the mechanisms of the action of DOX are not only selective to the tumor cells. They also cause several side effects, especially cardiotoxicity. Congestive heart failure occurs in 7%, 18%, and 65% of cancer patients treated with anthracyclines at cumulative doses of 150, 350, and 550 mg/m^2^, respectively [6]. Other side effects include hepatotoxicity, which affects 30.4% of patients undergoing breast cancer treatment [7], and nephrotoxicity, recently reported in patients with metastatic breast cancer [8]. Furthermore, hematological toxicities induced by DOX have already been reported in patients with these solid tumors, such as hemoglobinemia, leukopenia, and neutropenia [9,10]. The appearance of these complications can lead to changes in the chemotherapy or even its interruption [11]. The major mechanism related to DOX toxicity is the increase in oxidative stress and direct cytotoxicity due to the massive accumulation of reactive oxygen species (ROS) in cardiac [12], hepatic [13] and renal tissues [14]. Nonetheless, other mechanisms are still being investigated to understand better DOX toxicity in several organic systems [15].

The side effects caused by DOX, and the importance of this drug in cancer treatment, elicit the great need for interventions that allow its safe use. Preclinical studies with rodents have demonstrated the potential effectiveness of natural dietary supplements in softening or neutralizing these side effects, including interventions with minerals, vitamins, and polyunsaturated fatty acids [16]. Omega 3 (ω-3) is a long-chain polyunsaturated fatty acids (PUFAs) class that shows a double bond between the third and fourth carbon, counting from the terminal methyl group. The main PUFAs are alpha-linolenic acid (ALA 18:3), eicosapentaenoic acid (EPA 20:5) and docosahexaenoic acid (DHA 22:6) [17]. ALA is mainly found in nuts, seeds, and vegetable oils, while EPA and DHA are obtained by the intake of oily fish. PUFAs are integrated into the cell membrane (phospholipids), contributing to membrane fluidity and other cellular functions, such as inflammation and cell signaling [18]. Administration of ω-3 has already been shown to exert anti-inflammatory effects against DOX toxicity in cardiac tissue, as indicated by a reduction in a DOX-induced release of pro-inflammatory cytokines [19]. In rodent studies, a preventive supplementation with ω-3 [400 mg/kg body weight (bw)] for 28 days before the administration of a single dose of DOX (30 mg/kg bw) resulted in a reduction in oxidative stress, an increase in antioxidant enzymes and improved histological changes induced by DOX in the liver, kidneys, and testis [20,21]. However, most DOX administrations in humans are multidose regimens since previous investigations using single doses do not often provide the mechanistical insights of ω-3-mediated protection on hepatic and renal tissues.

Thus, this study aimed to evaluate the effects of ω-3 supplementation on hepatotoxicity and nephrotoxicity induced by repeated doses of DOX in male Wistar rats.

## 2. Results

### 2.1. The ω-3 Supplementation Attenuates a DOX-Induced Increase in Kidney Damage Markers

**Figure 1:**
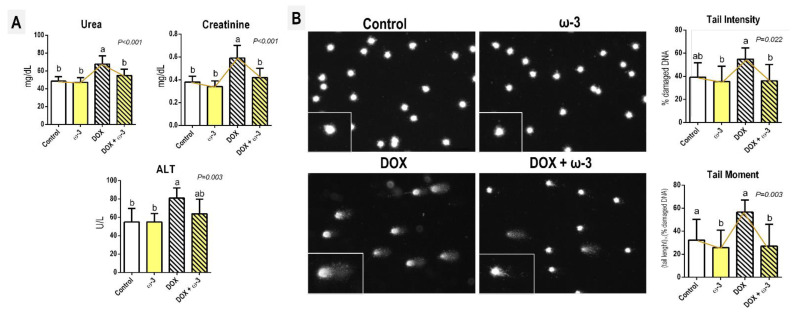
Effects of omega 3 (ω-3) supplementation on doxorubicin (DOX)-induced effects on (**A**) serum urea, creatinine, and alanine aminotransferase (ALT) levels; (**B**) peripheral blood genotoxicity. Representative photomicrographs of nucleoids are also presented. Data are presented as mean ± standard deviation. N = 5 (serum) or 8 (genotoxicity) rats/group. Male Wistar rats received ω-3 (400 mg/kg body weight/day) by intragastrical administrations for six weeks (or vehicle). After two weeks of ω-3 supplementation, animals received weekly single intraperitoneal injections of DOX (3.5 mg/kg body weight) for four weeks (or vehicle). The letters correspond to the statistical difference among groups by one-way ANOVA followed by a posteriori Tukey test (*p* < 0.05). Vectors (yellow) interconnecting the medians depict the statistical modulation of DOX-induced effects by ω-3.

The DOX regimen adopted led to a significant decrease in final body weight, body weight gain, liver weights (absolute and relative), and relative weight of left and right kidneys (*p* < 0.001, for all) (Table 1) compared to control counterparts, as a consequence of known systemic DOX-induced toxicity [13,22]. Although ω-3 attenuated the DOX-induced decrease in absolute liver weight (*p* < 0.001), this supplementation did not modify the DOX-related effects in general (Table 1). Regarding serum markers, DOX increased ALT (*p* = 0.003), urea and creatinine (*p* < 0.001, for both) levels. Both markers of kidney function/damage were reduced by ω-3 supplementation (*p* < 0.001) (Figure 1A).

### 2.2. The ω-3 Supplementation Attenuates a DOX-Induced Increase in Blood Genotoxicity

The DOX regimen adopted also led to a marked increase in peripheral blood genotoxicity compared to the control and/or ω-3 counterparts, as demonstrated by Tail Intensity (*p* = 0.022) and Tail Moment parameters (*p* = 0.03) (Figure 1B). Of note, ω-3 supplementation significantly reduced the DOX-induced increase in both parameters, indicating a reduction in DNA damage in peripheral blood cells (Figure 1B). However, the ω-3 supplementation alone did not alter any of the above-cited parameters (Table 1, Figure 1A,B), eliciting the safety of this short-term intervention.

### 2.3. The ω-3 Supplementation Reduced DOX-Induced Kidney Tubule Dilatation

**Figure 2:**
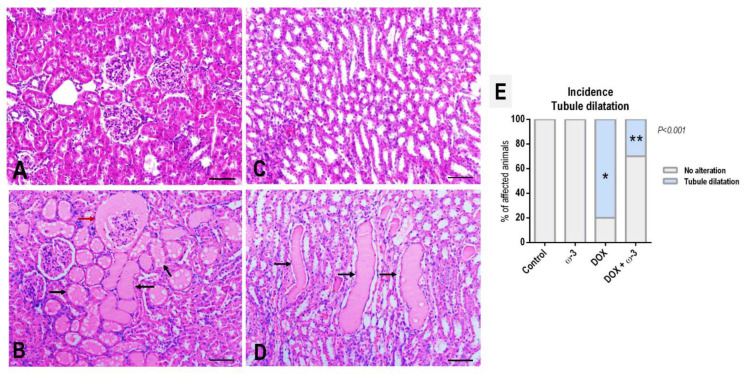
Representative photomicrographs of hematoxylin and eosin (HE)-stained sections of the (**A**,**B**) cortical and (**C**,**D**) medullar regions of the kidney of vehicle-(**A**,**C**) and (**B**,**D**) DOX-treated rats (scale bar: 100 µm). DOX-treated rats showed focal areas of tubular dilatation in both cortexes (black arrows), including Bowman’s space (red arrow) and medulla (black arrows), due to the appearance of hyaline (or eosinophilic proteinaceous) casts in the lumen of tubules. (**E**) Effects of omega 3 (ω-3) supplementation on doxorubicin (DOX)-induced effects on the incidence of kidney tubular dilatation. N = 10 rats/group. Male Wistar rats received ω-3 (400 mg/kg body weight/day) by intragastrical administrations for six weeks (or vehicle). After two weeks of ω-3 supplementation, animals received weekly single intraperitoneal injections of DOX (3.5 mg/kg body weight) for four weeks (or vehicle). * Statistical difference compared to the control group by Fisher’s Exact test (*p* < 0.05). ** Statistical difference compared to DOX group by Fisher’s Exact test (*p* < 0.05).

In HE-stained sections, histopathological analysis of the kidneys revealed focal areas of tubular dilatation in both cortexes (including the Bowman’s space) and medulla due to the appearance of hyaline (or eosinophilic proteinaceous) casts in the lumen of tubules—indicating an increased glomerular permeability often associated with glomerular damage [23]—as depicted in representative photomicrographs (Figure 2). The DOX regimen increased the incidence of these alterations, regardless of their manifestation (cortex and/or medulla), compared to the control group (*p* < 0.001). The ω-3 supplementation significantly reduced, by 50% (80% to 30%), the occurrence of this histological alteration in DOX-submitted rats (*p* < 0.001) (Figure 2).

**Figure 3:**
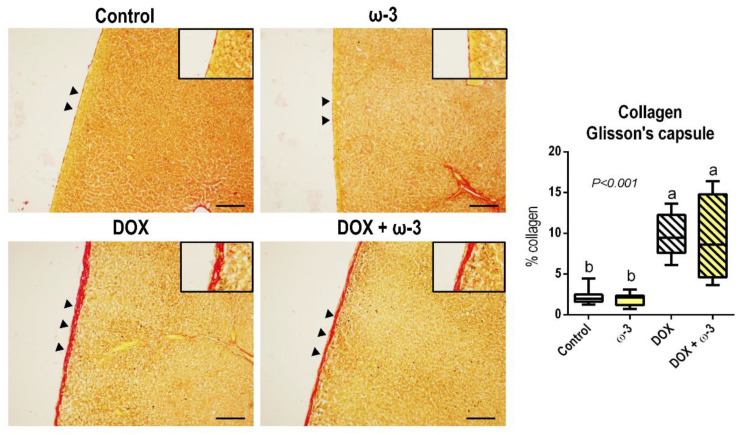
Effects of omega 3 (ω-3) supplementation on doxorubicin (DOX)-induced effects on collagen area (Sirius red-stained sections) in hepatic Glisson’s capsule (arrowheads). Representative photomicrographs are presented [scale bar: 100 µm] Data are presented as mean and standard deviation. N = 8 rats/group. Male Wistar rats received ω-3 (400 mg/kg body weight/day) by intragastrical administrations for six weeks (or vehicle). After two weeks of ω-3 supplementation, animals received weekly single intraperitoneal injections of DOX (3.5 mg/kg body weight) for four weeks (or vehicle). The letters correspond to the statistical difference among groups by Kruskal-Wallis followed by a posteriori Tukey test (*p* < 0.05).

No marked histopathological alterations were identified in liver parenchyma in response to DOX and/or ω-3 interventions after a histopathological analysis of the HE-stained sections (Supplementary Data S1). Nonetheless, a thickening of hepatic Glisson’s fibrous capsule was observed in Sirius-red stained sections in both DOX-treated groups (*p* < 0.001) (Figure 3) in response to DOX-induced peritonitis, which is a known side effect of this drug in rats [24]. Representative photomicrographs of HE-sections showing capsule thickening are also shown in Supplementary Data S1. DOX also led to a slight but significant increase in collagen in the portal areas (*p* < 0.001), although no marked fibrous expansions were observed. DOX also tended to increase collagen in the stroma of the kidney cortex and medulla (Supplementary Data S2). The ω-3 supplementation did not alter these DOX-induced effects in the liver and kidneys (Figure 3, Supplementary Data S2). The animals supplemented with ω-3 alone for 6 weeks showed no histopathological alterations, reinforcing the safety of this intervention on these organs.

### 2.4. The ω-3 Supplementation Reduced a DOX-Induced Increase in Hepatocyte Proliferation

**Figure 4:**
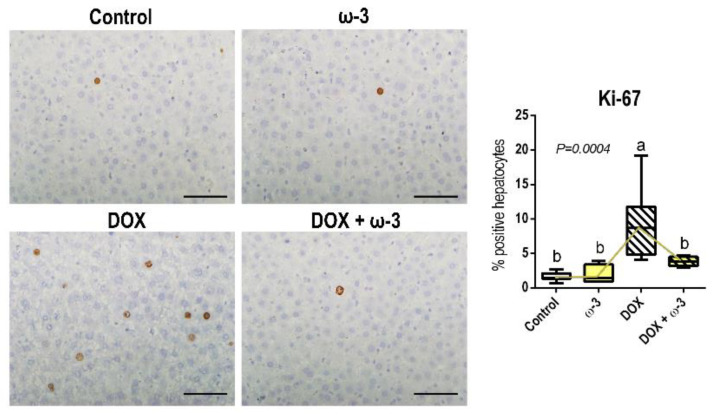
Effects of omega 3 (ω-3) supplementation on doxorubicin (DOX)-induced effects on hepatocyte proliferation (Ki-67). Representative photomicrographs are presented [scale bar: 40 µm] Data are presented as box plots. N = 7 rats/group. Male Wistar rats received ω-3 (400 mg/kg body weight/day) by intragastrical administrations for six weeks (or vehicle). After two weeks of ω-3 supplementation, animals received weekly single intraperitoneal injections of DOX (3.5 mg/kg body weight) for four weeks (or vehicle). The different letters correspond to the statistical difference among groups by Kruskal-Wallis followed by a posteriori Tukey test (*p* < 0.05). Vectors (yellow) interconnecting the medians depict the statistical modulation of DOX-induced effects by ω-3.

The DOX regimen adopted increased hepatocyte proliferation (*p* = 0.0004), as demonstrated by Ki-67 immunostaining (Figure 4). The ω-3 supplementation was able to revert this DOX-mediated indirect effect on the liver (Figure 4), which may be triggered as a compensatory response to DOX-induced hepatocyte damage, as indicated by increased ALT serum levels. Both DOX and ω-3 did not affect cell proliferation of tubular epithelial cells in the kidney cortex and medulla (Supplementary Data S3).

### 2.5. The ω-3 Supplementation Reduced a DOX-Induced Increase in Hepatic p65 Protein

**Figure 5:**
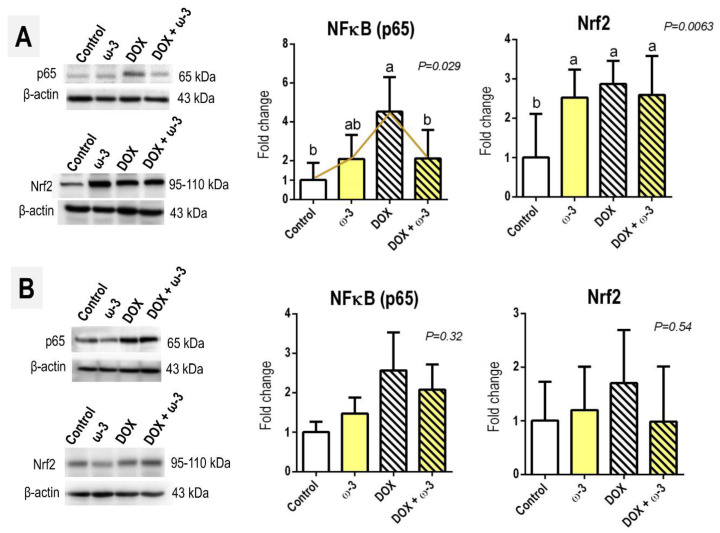
Effects of omega 3 (ω-3) supplementation on doxorubicin (DOX)-induced effects p65 and Nrf2 protein levels in the (**A**) liver and (**B**) kidney. Representative blot bands are presented. Data are presented as mean and standard deviation. N = 6 rats/group. Male Wistar rats received ω-3 (400 mg/kg body weight/day) by intragastrical administrations for six weeks (or vehicle). After two weeks of ω-3 supplementation, animals received weekly single intraperitoneal injections of DOX (3.5 mg/kg body weight) for fr weeks (or vehicle). The different letters correspond to the statistical difference among groups by one-way ANOVA followed by a posteriori Tukey test (*p* < 0.05). Vectors (yellow) interconnecting the medians depict the statistical modulation of DOX-induced effects by ω-3.

The DOX protocol adopted led to a 4-fold increase in the protein levels of the hepatic p65 subunit with a pro-inflammatory transcription factor NFκB—which is also closely related to a damage/inflammation-induced hepatocyte survival/proliferative response [25]—and this effect was attenuated by ω-3 intervention (*p* = 0.029) (Figure 5). However, the ω-3 supplementation could not modify the DOX-induced increase in hepatic Nrf2 protein levels (*p* = 0.0063), which may have been elevated in response to increased drug-induced oxidative stress. Although both p65 and Nrf2 had increased slightly in the kidney by DOX, the groups were not statistically different (Figure 5).

## 3. Discussion

Our preclinical bioassay evaluated the effects of ω-3 supplementation on the DOX-induced toxic impact on the kidney and liver of male Wistar rats. In contrast to other recent rodent bioassays based on a single DOX administration [26,27,28], our design was based on multiple i.p. doses, resembling first-line DOX-based human chemotherapy regimens [29,30]. In brief, DOX increased systemic genotoxicity and caused liver-related (increase in serum ALT levels, Glisson’s capsule thickness, compensatory proliferation and p65 levels) and kidney-related (increased serum urea and creatinine levels, and incidence of tubular dilatation) deleterious outcomes. Considering that irreversible cardiotoxicity and gonadotoxicity are addressed as the main side effects of DOX therapy [5,31,32], liver and kidney outcomes are usually neglected or poorly investigated/reported in clinical practice. Since these are essential organs for DOX metabolism and clearance, our findings reinforce the importance of screening DOX-induced hepatotoxicity and nephrotoxicity.

In contrast to these effects, ω-3 supplementation was safe and abrogated the DOX-related enhancement of genotoxicity, urea and creatinine. Furthermore, ω-3 intervention reduced the incidence of tubular dilatation in the kidney while it reduced p65 protein levels and the toxicity-induced proliferative response in the liver. Preclinical studies investigating possible approaches towards mitigating cardiotoxicity have been significantly encouraged recently [33,34,35,36]. Our preclinical findings on ω-3 may inspire future clinical studies on attenuating kidney and liver outcomes.

Regarding liver-related outcomes, after four weeks of DOX administrations, DOX-treated rats developed a thickened fibrous capsule and increased collagen in the portal areas, similar to previous findings in mice [37]. Glisson’s capsule thickening is attributed to DOX-induced peritonitis [24], while increased collagen in vascular structures may be associated with the pharmacokinetics of this anthracycline. DOX is predominantly metabolized by the hepatocytes, after entering (by diffusion) the portal system, to the major metabolite, the quinone doxorubicinol (DOXOL), and several cytotoxic aglycone metabolites. However, most of the DOX administrated (~50%) is excreted from the body into the bile without being biotransformed [38,39,40]. In the hepatocytes, DOXOL generation, which is catalyzed by the enzyme nicotinamide adenine dinucleotide phosphate hydrogen (NADPH) reductases, leads to the production of reactive oxygen species (ROS), such as superoxide radicals and secondary reactive species, as lipid hydroperoxides [40]. These compounds cause direct hepatic damage and death, culminating in the leakage of hepatic enzymes, such as ALT [41]. Hepatocyte death triggers an increase in p65 levels and compensatory proliferation, as indicated by higher Ki-67 indices. In addition, DOX-mediated oxidative stress activates Nrf2-mediated antioxidant system response, as shown by increased protein levels of this transcription factor. In general, this DOX-mediated background impairs the normal energetic metabolism of hepatic tissue [41], which could lead to decreased body weight gain.

In contrast to these hepatotoxic effects, ω-3 reduced the hepatic p65 levels and hepatocyte proliferation, although this supplement did not modify hepatic Nrf2 levels. According to the Human Equivalent Dose (HED), using an allometric dose translation [42], our 400 mg/kg bwt ω-3 intervention is equivalent to the human dose of 65 mg/kg bwt, or a ~4 g daily dose for 60 kg humans, that equals 4 capsules of the commercial formulation used in our study. As briefly mentioned, p65 pathway activation is related to a pro-inflammatory response and stimulates a hepatocyte survival/proliferative response under damage or inflammation [25]. The ω-3 supplementation seems to negatively modulate downstream inflammatory responses mediated by NFκB in different tissues under injury stimuli, including the liver [43,44]. Previous findings indicate that ω-3 alleviates p65-induced inflammation through a Sirt1-mediated mechanism in hepatocytes. The nuclear translocation of nuclear p65 was significantly decreased after ω-3 treatment, while this effect was reversed after treatment with EX-527, a Sirt1 inhibitor [43]. p65 attenuation may reduce its downstream responses, including the proliferative stimuli. Considering that the development of therapy-related malignant neoplasms involving a sustained cell proliferation hallmark is of the most deleterious DOX side effects [5], this ω-3 effect may also be beneficial in this aspect, although further investigation is required.

DOX and its reactive metabolites reach the systemic circulation after the first pass in the liver. Then it reaches the other organs. Therefore, monitoring DOX and DOX-OL levels in circulation is proposed to indicate DOX toxicity for cancer patients [45]. We found that DOX enhanced peripheral blood DNA damage, indicating increased systemic genotoxicity in response to DOX-induced oxidative stress. On the other hand, ω-3 supplementation decreased this DOX-mediated effect. In a similar bioassay, a preventive ω-3 supplementation (400 mg/kg bwt/day) for 30 days decreased the serum oxidative stress (malondialdehyde) induced by a single DOX administration while increasing serum reduced glutathione levels [21]. As such, in addition to DOX and DOX-OL determination in circulation, genotoxicity assessment in DOX bioassays should also be encouraged, enabling the early screening of potential preventive treatments.

Regarding kidney-related effects, DOX led to increased serum creatinine and urea levels and an augmented incidence of tubular dilatation due to eosinophilic proteinaceous casts. Nonetheless, DOX did not alter renal collagen deposition, epithelial cell proliferation and Nrf2/p65 levels in this organ. Although most DOX is excreted via bile, some DOX and its metabolites reach the kidneys and are excreted through the urinary system [46]. The serum and histopathological alterations indicate increased glomerular permeability often associated with glomerular damage [23]. Indeed, other studies suggest that DOX-induced oxidative stress and free radical formation of ferric anthracycline can be highly responsible for this direct nephrotoxic effect [47]. The supplementation of ω-3 reduced creatinine, urea levels and tubular dilatation occurrence. This effect does not seem to be associated with Nrf2 and p65 transcription factors, as they were unmodified by ω-3 intervention, although glomerular permeability-related mechanisms still need investigating.

The main limitation of our study, along with other recent bioassays, is that an experimental model has not been included in which DOX was used to treat animals with preexisting cancer [34]. Studies on the combined antitumoral effects of omega-3 and DOX are scarce, but ω-3 PUFA was shown to enhance DOX antitumoral effects in vivo (a MatBIII mammary adenocarcinoma tumor-bearing rat model) and in vitro (human A549 lung carcinoma and MCF-7 breast adenocarcinoma cell lines), partly by enhancing DOX concentration [48,49]. Moreover, as DOX is known to induce bone marrow suppression, the roles of this hematotoxic outcome on DOX-induced liver and kidney effects should be further explored, as the administration of bone marrow-derived mesenchymal stem cells alleviated DOX-induced liver injury in rats by improving the oxidative status and limiting apoptotic cell death [50]. Our findings indicate that our preclinical translatable ω-3 intervention attenuated DOX-induced deleterious effects on the liver and kidney. In addition to these beneficial effects, ω-3 supplementation is considered safe since this intervention had no marked biochemical, morphological, or molecular outcomes in both organs in the absence of DOX treatment. Therefore, our findings may inspire future mechanistical investigations and clinical interventions.

## 4. Materials and Methods

### 4.1. Experimental Design

**Figure 6:**
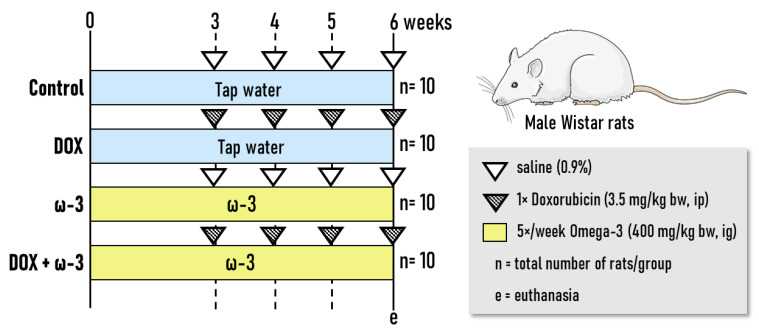
Experimental design. This figure was composed with the aid of SMARTServier free.

Seven-week-old male Wistar rats (*n* = 40 animals) were randomly allocated into four groups (*n* = 10 rats/group, Figure 6). Initially, the ω-3 and DOX + ω-3 groups were treated with ω-3 [400 mg/kg of bw/day, Essential Nutrition, São José, Brazil] [20,21] by intragastrical (ig) administration for six weeks, while other groups received tap water (Figure 1). Doses of ω-3 were based on a previous rat bioassay showing cardioprotective effects against DOX-induced cardiotoxicity [20]. ω-3 contained (each gram): 770 mg of total fats; 115 mg of saturated fats, 154 mg of monounsaturated fats; 461 mg of polyunsaturated fats; 277 mg of EPA; 185 mg of DHA; 1.15 mg of cholesterol and 1.15 mg of Vitamin E.

After two weeks of ω-3 supplementation, animals from DOX and DOX + ω-3 groups received weekly single intraperitoneal (ip) injections of DOX (3.5 mg/kg bw) for four weeks (cumulative dose of 14 mg/kg bw) (Figure 6). Doses of DOX were based on Xue et al. [49] and Spivak et al. [51] regimens, given that the cumulative dose is the maximally tolerated dose for rats. Therefore, ω-3 was administered before (2 weeks) and during (4 weeks) the DOX regimen. After 6 weeks, animals were euthanized per decapitation under anesthesia (sodium thiopental, 120 mg/kg, ip). Blood samples were collected and centrifuged (1500× *g*, 10 min), and the serum was stored at −20 °C for biochemical assays. During necropsy, the liver and kidneys were removed, washed in saline solution 0.9% and weighed. Liver samples (lateral left lobe) and slices from both kidneys were collected and fixed in buffered formalin 10% or stored at −80 °C. After fixation, samples from both organs were kept in 70% alcohol until histological processing. Animals received drinking water and chow (Nuvital, Nuvilab, Brazil) ad libitum. In addition, animals were kept in propylene cages with stainless steel covers and pine wood shavings for bedding in a room that had continuous ventilation (16–18 air changes/hour), relative humidity (55 ± 10%), controlled temperature (22 ± 2 °C) and light/dark cycle 12:12 h. Body weight, water and food consumption, and the condition of the animals’ health were all monitored and recorded twice a week. This animal study was performed under the approval of the Botucatu Medical School (FMB/UNESP) Ethics Committee on Use of Animals (CEUA) (1295/2019). All animals received humane care according to the criteria described in the “Guide for the Care and Use of Laboratory Animals” [52].

### 4.2. Genotoxicity Assessment

Four hours after the last injection of DOX, peripheral blood samples were collected from the periorbital plexus for the genotoxicity assessment using alkaline single cell gel electrophoresis (comet) assay, as previously established [53]. Blood samples were quickly mixed with low melting point agarose (100 µL 0.75% in PBS, Invitrogen, Waltham, MA, USA) and spread onto slides pre-coated with normal point agarose (1.5% in PBS, Invitrogen, Waltham, MA, USA) and covered with coverslips. Following agarose solidification (4 °C for 5 min), coverslips were carefully removed. The slides were incubated with cold lysis solution (2.5 M NaCl, 100 mM Na2EDTA, 10 mM Tris–HCl, 1% N-lauroyl-sarcosine, 1% Triton X-100 and 10% DMSO, pH 10) overnight, at 4 °C. Subsequently. The slides were washed three times in PBS and immersed in a fresh cold alkaline electrophoresis buffer (300 mM NaOH, 1 mM Na_2_EDTA, pH > 13) for 20 min. Electrophoresis was conducted for 20 min at 1 V/cm (300 mA) for 20 min. The slides were then neutralized with 0.4 M Tris (pH 7.5), dehydrated in 100% ethanol, and stained with SYBR Gold solution (1:10,000) (Invitrogen, USA). Fifty randomly selected nucleoids were scored in each slide (two slides/animal) under an epi-fluorescence microscope (Olympus BX-50, Shinjuku, Japan) using Comet Assay IV software (Perceptive Instruments, Great Shelford, UK). Tail intensity (% of DNA in comet tail) and Tail moment (% of DNA in comet tail/tail length) was chosen as reliable parameter to evaluate DNA damage [54].

### 4.3. ALT, Creatinine and Urea Serum Determination

Serum samples (−20 °C) were used to evaluate alanine aminotransferase (ALT) enzyme levels (i.e., hepatic damage), creatinine and urea (i.e., kidney function) using the colorimetric enzymatic method with the use of commercial kits (BioClin, Belo Horizonte, Brasil) in an automatic spectrophotometric analyzer (Chemistry Analyzer Bs-200, Mindray Medical International Limited, Shenzhen, China).

### 4.4. Histopathological Evaluation and Collagen Morphometry

Formalin-fixed liver and kidney samples were embedded in paraffin blocks, and 5 µm sections were obtained and stained with hematoxylin and eosin (HE) or Sirius Red. Histopathological alterations in both organs were identified in a section stained with HE, following the criteria established in the literature [23,55]. The incidence (% lesion-bearing rats/group) was evaluated in all experimental groups. Collagen morphometry was analyzed in Sirius red-stained sections. Kidney (cortex and medulla) and liver (portal areas and Glisson’s capsule) were randomly photographed (40× objective, 10 fields/slide/rat), and the collagen area was calculated using Leica QWin 3.0 software (Leica Microsystems, Wetzlar, Germany).

### 4.5. Immunohistochemistry for Ki-67

Other histological slides (5 μm) were obtained on silane-covered microscope slides. Then, slides were subjected to antigen retrieval in 0.01 M citrate buffer (pH 6.0, 5 min, 120 °C) in a Pascal Pressure Chamber (Dako Cytomation, Glostrup, Denmark). After endogenous peroxidase blockade with 10% H_2_O_2_ in phosphate-buffered saline (PBS) (15 min.), the slides were treated with skim milk (60 min.) and incubated with anti-Ki-67 (i.e., a cell proliferation marker, MA5-14520, 1:100 dilution, primary antibody in a humidified chamber (overnight, 4 °C). Then, the slides were incubated with a one-step horseradish peroxidase (HRP)-polymer (EasyPath—Erviegas, Indaiatuba, Brazil) (20 min). The reaction was visualized with 3′3-diaminobenzidine (DAB) chromogen (Sigma Aldrich, Burlington, MA, USA) and counterstained with Harris’ hematoxylin. A semiquantitative analysis was comprised of calculating the immunolabelling index (%) and counting Ki-67-positive and negative hepatocytes and kidney epithelial cells (cortex and medulla) in 20 randomly selected fields (40× objective, 1 section/animal), as obtained using Olympus cellSens software (Olympus Corporation, Shinjuku, Japan).

### 4.6. Immunoblot

Liver and kidney samples (~100 mg) were homogenized in RIPA buffer (Sigma-Aldrich, USA) containing a 1% protease inhibitor cocktail (Sigma-Aldrich, Burlington, MA, USA) (4 °C for 2 h). Protein samples were heated (95 °C, 5 min) in a Laemmli sample buffer and then electrophoretically separated in a 10% SDS–PAGE gel under reducing conditions and transferred to nitrocellulose membranes (Bio-rad Laboratories, Hercules, CA, USA). Membranes were blocked with skim milk in Tris-Buffered Saline-Tween (TBS-T, 1 h). Membranes were subsequently incubated using anti-p65-NFκB (sc-372, 65 kDa, 1:1000 dilution, Santa Cruz Biotechnology, Dallas, TX, USA), anti-Nrf2 (PA5–27882, ~95–110 kDa, 1:1000, Thermo Fisher Scientific, Waltham, MA, USA), or anti-β-actin (4970S, 43 kDa, 1:1000 dilution, Cell Signaling, Danvers, MA, USA) primary antibodies were diluted in TBS-T overnight. Membranes were incubated with specific HRP-conjugated secondary antibodies according to the primary antibodies used (2 h). Finally, protein signals were detected using Clarity Max ECL Substrate (Bio-Rad Laboratories, Hercules, CA, USA). Signals were captured by a G: BOX Chemi system (Syngene, Cambridge, UK) controlled by automatic software (GeneSys, Syngene, Cambridge, UK). Band intensities were quantified using densitometry analysis Image J software (NIH, Bethesda, MD, USA). Finally, protein levels were reported as a fold change according to β-actin protein expression.

### 4.7. Statistical Analysis

Data were analyzed by one-way analysis of variance (ANOVA) or Kruskal-Wallis and a posteriori Tukey test (*p* < 0.05). Statistical analyses were performed using GraphPad Prism software 6.0 (GraphPad, San Diego, CA, USA). Data are presented as mean + standard deviation (S.D.) or box plots. The number of replicates (*n*) per group for each analysis is displayed in figure/table captions.

## Figures and Tables

**Table 1:** Effects of omega 3 (ω-3) supplementation on doxorubicin (DOX)-induced effects on body weight parameters and liver and kidney weights (absolute and relative).

Parameters	Groups/Treatments				*p* Value
	Control	ω-3	DOX	DOX + ω-3	
N	10	10	10	10	
Initial (g)	279.18 ± 25.47	278.1 ± 22.23	280.67 ± 30.56	279.5 ± 24.55	=0.997
Final (g)	412.04 ± 40.29 a	416.05 ± 36.52 a	283.21 ± 43.07 b	314.9 ± 30.91 b	<0.001
Gain (g)	132.85 ± 19.38 a	137.95 ± 17.28 a	1.11 ± 37.2 b	35.4 ± 27.28 b	<0.001
Absolute liver weight (g)	13.69 ± 1.14 a	14.26 ± 1.68 a	10.84 ± 2.34 b	13.11 ± 1.13 a	<0.001
Relative liver weight (%)	3.33 ± 0.15 a	3.42 ± 0.25 a	3.81 ± 0.46 b	4.18 ± 0.36 b	<0.001
Absolute right kidney weight (g)	1.44 ± 0.11	1.35 ± 0.14	1.23 ± 0.34	1.24 ± 0.21	=0.107
Relative right kidney weight (%)	0.35 ± 0.04 a	0.32 ± 0.02 a	0.44 ± 0.10 b	0.39 ± 0.05 ab	<0.001
Absolute left kidney weight (g)	1.46 ± 0.15 a	1.34 ± 0.14 ab	1.15 ± 0.47 b	1.26 ± 0.19 ab	=0.027
Relative left kidney weight (%)	0.35 ± 0.03 a	0.32 ± 0.02 a	0.41 ± 0.15 b	0.40 ± 0.05 b	<0.001

Data presented as mean ± standard deviation. N = the number of rats/group. Wistar rats received ω-3 (400 mg/kg body weight/day) by intragastrical administrations for six weeks (or vehicle). After two weeks of ω-3 treatment, animals received weekly single intraperitoneal injections of DOX (3.5 mg/kg body weight) for four weeks (or vehicle). The letters correspond to the statistical difference among groups by one-way ANOVA followed by a posteriori Tukey test (*p* < 0.05).

## Data Availability

All relevant data are presented in the manuscript. Raw data will be provided upon reasonable request.

## Supplementary Materials

The following supporting information can be downloaded at: https://www.mdpi.com/article/10.3390/molecules28073004/s1, Supplementary Data S1: Representative photomicrographs of HE-stained sections (scale bar: 50 µm) of the (**A**) hepatic parenchyma and (**B**) Glisson’s capsule (arrowheads). Wistar rats received omega (ω-3, 400 mg/kg body weight/day) by intragastrical administrations for six weeks (or vehicle). After two weeks of ω-3 supplementation, animals received weekly single intraperitoneal injections of doxorubicin (DOX, 3.5 mg/kg body weight) for four weeks (or vehicle). Supplementary Data S2: Effects of omega 3 (ω-3) supplementation on doxorubicin (DOX)-induced effects on collagen area (Sirius red-stained sections) in hepatic portal areas, kidney cortex and medulla. Representative photomicrographs are presented [scale bar: 20 (liver) or 50 (kidney) µm]. Data are presented as box plots. N = 8 rats/group. Wistar rats received ω-3 (400 mg/kg body weight/day) by intragastrical administrations for six weeks (or vehicle). After two weeks of ω-3 supplementation, animals received weekly single intraperitoneal injections of DOX (3.5 mg/kg body weight) for four weeks (or vehicle). The different letters correspond to the statistical difference among groups by Kruskal-Wallis followed by a posteriori Tukey test (*p* < 0.05). Supplementary Data S3: Effects of omega 3 (ω-3) supplementation on doxorubicin (DOX)-induced effects epithelial cell proliferation (Ki-67-immunostained sections) in kidney cortex and medulla. Representative photomicrographs are presented [scale bar: 20 µm]. Data are presented as box plots. N = 7 rats/group. Wistar rats received ω-3 (400 mg/kg body weight/day) by intragastrical administrations for six weeks (or vehicle). After two weeks of ω-3 supplementation, animals received weekly single intraperitoneal injections of DOX (3.5 mg/kg body weight) for four weeks (or vehicle). Data were analyzed by Kruskal-Wallis followed by a posteriori Tukey test (*p* < 0.05).

## Sample Availability

Samples of the compounds are commercially available.
