# Supplementary material for: Protective Effects of Omega-3 Supplementation against Doxorubicin-Induced Deleterious Effects on the Liver and Kidneys of Rats
Source: Molecules. 2023 Mar 28;28(7):3004. doi: 10.3390/molecules28073004 (PMC10096317; doi:10.3390/molecules28073004)
Supplement: Supplementary file 1 [file molecules-28-03004-s001.zip › Supplementary Data S1 - HE liver.pdf]

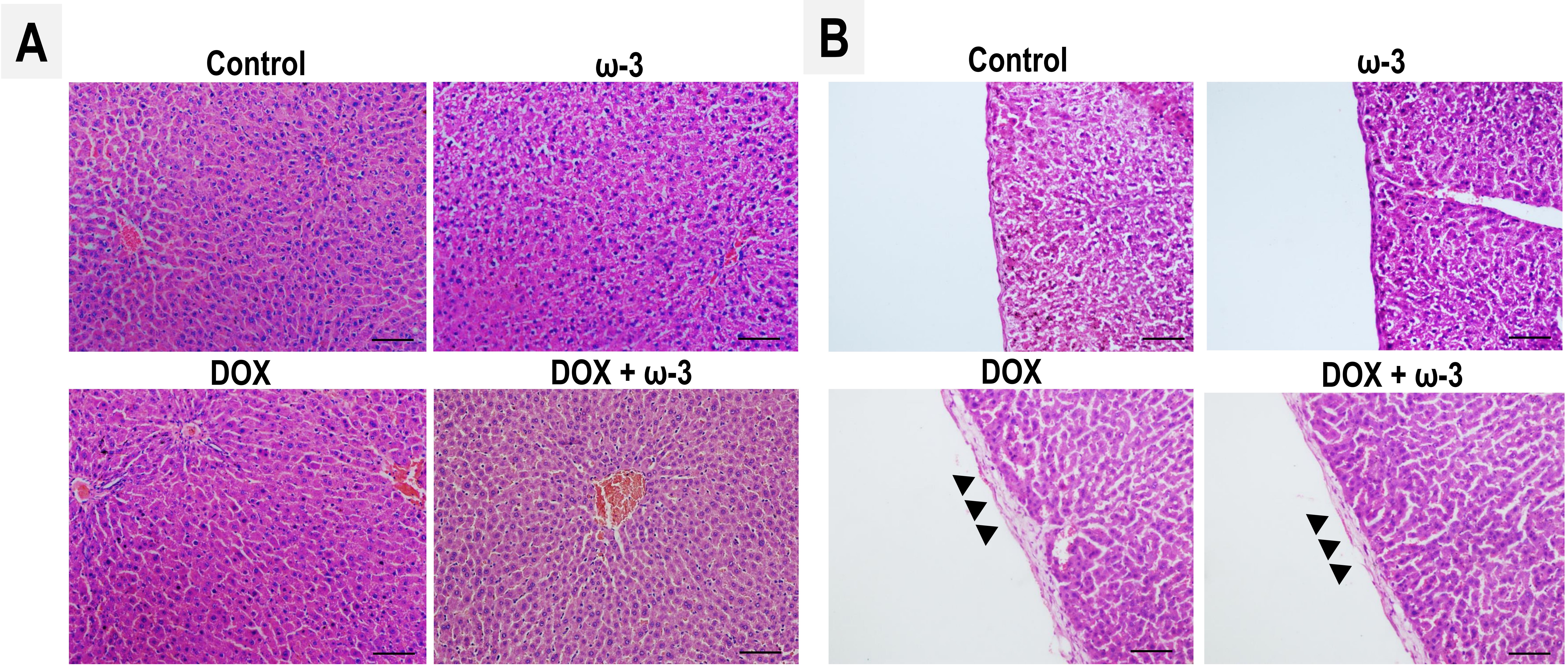

Supplementary Data S1. Representative photomicrographs of HE-stained sections (scale bar: 50 μm) of the **(A)** hepatic parenchyma and **(B)** Glisson's capsule (arrowheads). Wistar rats received omega (ω-3, 400 mg/kg body weight/day) by intragastrical administrations for six weeks (or vehicle). After two weeks of ω-3 supplementation, animals received weekly single intraperitoneal injections of doxorubicin (DOX, 3.5 mg/kg body weight) for four weeks (or vehicle).
