# Supplementary material for: Protective Effects of Omega-3 Supplementation against Doxorubicin-Induced Deleterious Effects on the Liver and Kidneys of Rats
Source: Molecules. 2023 Mar 28;28(7):3004. doi: 10.3390/molecules28073004 (PMC10096317; doi:10.3390/molecules28073004)
Supplement: Supplementary file 1 [file molecules-28-03004-s001.zip › Supplementary Data S3 - Ki67 kidney.pdf]

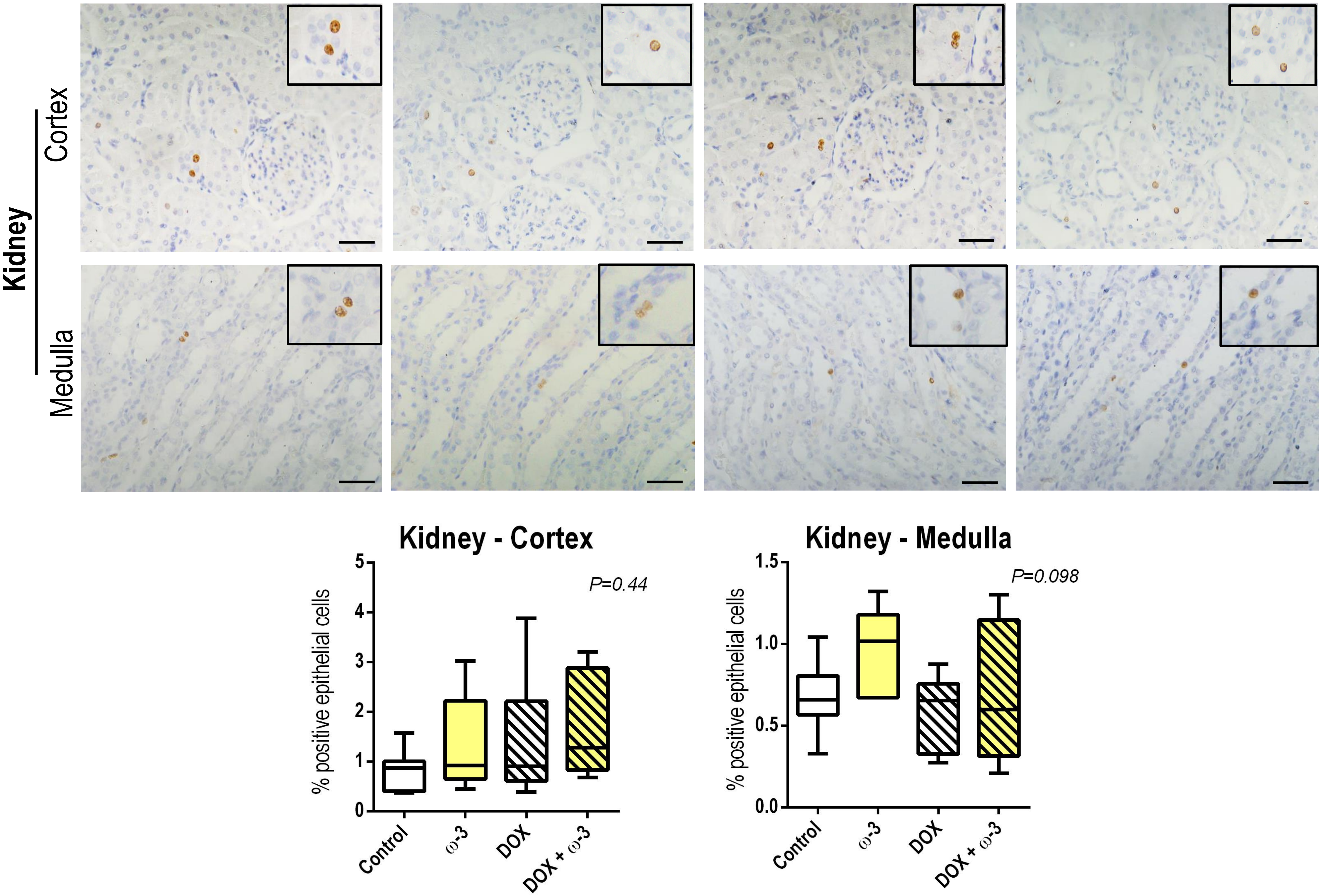

Supplementary Data S3. Effects of omega 3 (ω-3) supplementation on doxorubicin (DOX)-induced effects epithelial cell proliferation (Ki-67-immunostained sections) in kidney cortex and medulla. Representative photomicrographs are presented [scale bar: 20 µm]. Data are presented as box plots. N = 7 rats/group. Wistar rats received ω-3 (400 mg/kg body weight/day) by intragastrical administrations for six weeks (or vehicle). After two weeks of ω-3 supplementation, animals received weekly single intraperitoneal injections of DOX (3.5 mg/kg body weight) for four weeks (or vehicle). Data were analyzed by Kruskal-Wallis followed by *a posteriori* Tukey test (*P<0.05*).
